# Editorial: The Role of the Gut Microbiota in Health and Inflammatory Diseases

**DOI:** 10.3389/fimmu.2020.565305

**Published:** 2020-09-17

**Authors:** Ashutosh K. Mangalam, Javier Ochoa-Repáraz

**Affiliations:** ^1^Department of Pathology, University of Iowa, Iowa City, IA, United States; ^2^Department of Biology, Eastern Washington University, Cheney, WA, United States

**Keywords:** microbiota, immunomodulation, gut - associated lymphoid tissues (GALT), inflammation, autoimmunity, microbiome

Soon after birth, humans are colonized with a vast number of microbes, collectively termed the microbiota. Among the microbiota, commensal bacteria help shape and regulate a number of the host's physiological processes, including the immune system. Alterations in the gut microbial community (dysbiosis) have been linked with multiple diseases. Therefore, the identification of commensal bacteria that are depleted or enriched in the context of pathological conditions, and the mechanisms by which they predispose or protect from disease is an active area of research. In this collection, several articles discuss the biological factors and processes that are regulated by gut microbes, and the potential use of commensal bacteria as therapeutic agents to treat disease.

The diet of an individual has emerged as the strongest factor that influences the composition and function of the gut microbiota (1). The relevance of dietary factors in modulating the microbiota to maintain the immune response is highlighted by current evidence linking gut dysbiosis with multiple diseases (Yap and Marino). Although the contributions of commensal bacteria to T and B cell function have been studied extensively, its effect on intraepithelial lymphocytes (IELs) is poorly defined. Yap and Mariño discuss the current knowledge on the regulation of IELs by gut bacteria whereas Chen et al. use of antibiotic treatment and germ-free (GF) mice to illustrate the importance of commensal bacteria in regulating the function of IELs against gut microbes. In this study, the authors showed that in the absence of microbes, there is a significant reduction of CD8ab^+^ IELs, which impacts their ability to produce anti-microbial peptides (Chen et al.). Although the data are currently limited, there is evidence to suggest that the gut microbiota influences the function of IELs.

In a healthy individual, immune-tolerance is maintained by balancing levels of anti-inflammatory CD4^+^FoxP3^+^ regulatory T cells (Tregs) and proinflammatory Th17 cells. Certain commensals such as segmented filamentous bacteria (SFB) can induce Th17 cells (2), whereas others such as *Clostridium* species (3), *Bacteroides fragilis* (4), and *Prevotella histicola* (5) induce Tregs. Dysbiosis due to either reduced Treg induction and/or increased Th17 induction, can promote proinflammatory conditions, which can predispose individuals to, or exacerbate, disease (6). The implications of generating immunotolerance by inducing tolerogenic intestinal antigen-presenting cells, Tregs, and immunoregulatory cytokines have been discussed (Vitetta et al.). In this special issue, two studies utilized a model of subacute ileitis to decipher the role of the human microbiome in intestinal inflammation, and in extra-intestinal and systemic inflammation (Bereswill et al.; Heimesaat et al.). Both studies utilized a human microbiota-associated (hma) mouse model, in which abiotic mice were colonized with human feces and orally infected with a low-dose of *Toxoplasma gondii* to induce subacute ileitis. Using this model, Heimesaat et al. show the relevance of ileum acquired multidrug-resistant (MDR) *Pseudomonas aeruginosa* in causing systemic inflammation. Bereswill et al. utilize the hma model to show the therapeutic ability of the neuropeptide, pituitary adenylate cyclase-activating polypeptide, to treat subacute ileitis. These two studies highlight the relevance of using this unique model system to understand the pathogenesis of intestinal and systemic inflammatory diseases and its usefulness in testing novel therapies.

As the colon harbors the maximum density of commensal bacteria, the association of gut dysbiosis with colon pathology is not surprising. Okamoto et al. reported a patient with Cap polyposis (a rare heredity disease of the colon) successfully treated with antibiotics. The treatment with antibiotics caused the improvement in disease, that was associated with a significant change in the composition of the gut microbiota. Thus, the enrichment of pathobionts that are associated with gut dysbiosis is an emerging common theme among intestinal and extra-intestinal/systemic diseases. Restoring the microbial composition to a healthy state, either directly using gut commensal bacteria as therapeutic agents, or indirectly using chemical drugs (e.g., antibiotics), can significantly alleviate symptoms associated with these diseases.

Several manuscripts published previously and in this special issue report the use of gut commensal bacteria (single-strain or probiotic mixture) as a therapeutic option to correct dysbiosis and thereby treat disease (Maddaloni et al.; Shahi et al.; Swartwout and Luo; Vitetta et al.) As gut dysbiosis shifts the balance toward inflammatory, as opposed to immunoregulatory cell subsets, treatment with commensal bacteria that induce immunomodulation can restore homeostasis. Shahi et al. describe in their work that the human commensal bacteria, *Prevotella histicola*, is as effective as the MS drug Copaxone in suppressing disease in experimental autoimmune encephalomyelitis (EAE), an animal model of MS. Of note, the use of *Prevotella* is of particular relevance to human MS as it is decreased in abundance in untreated MS patients (7, 8). Correspondingly, the treatment of MS with disease-modifying therapies restores levels of *Prevotella* (9).

Maddaloni et al. describe the use of a genetically-engineered *Lactococcus lactis* designed to provide protection from autoimmune diseases such as arthritis by producing enhanced levels of IL-35. Using a murine model of collagen-induced arthritis, the authors show that oral treatment with IL-35-producing *L. lactis* induced protective responses by promoting the generation of IL-10-producing Tregs and decreasing interferon (IFN)-gamma and IL-17-mediated inflammation. Zhang et al. report that the human commensal *Bacteroides fragilis* protects from antibiotic-associated diarrhea by restoring the gut microbiota's composition to a healthy state. In addition to these studies, a *B. fragilis* strain capable of expressing the capsular polysaccharide A (PSA), has been shown to possess therapeutic potential against experimental models of ulcerative colitis (10), CNS demyelinating disease (11), asthma (12), and autism spectrum disorders (13). Thus, these studies highlight the therapeutic potential of both un-manipulated (*P. histicola* and *B. fragilis*) as well as engineered human commensals (*L lactis)* to treat various intestinal (antibiotic-induced diarrhea) and extra-intestinal diseases (MS and RA).

The administration of probiotics, prebiotics, or phages can provide health benefits by promoting a balanced immunological response. This microbial-based treatment may be especially relevant during pregnancy, as pregnancy significantly alters the immune system. The concept of maternal probiotics as a mechanism to modify the maternal-infant interface and promote immune homeostasis is attractive. Swartwout and Luo highlight the effects of pregnancy on the composition of the intestinal bacteria in the context of a broader discussion on the current lack of data regarding maternal probiotics and their effects on the incidence and progression of autoimmune diseases. Yet the excitement for this is limited, as additional experiments that test the approach's safety and efficacy are required.

The role of the microbiota in the generation of food allergies is an area of active research (14). One hypothesis is that food allergies are triggered by tolerance breakdown at the intestinal level (Berni Canani et al.), suggesting that understanding the role of the microbiota in breaking tolerance to diet could provide novel avenues to control food allergies. Pascal et al. propose that dysbiosis is a factor that triggers allergies, not only in the context of the intestinal milieu and food allergies but also in the skin and respiratory tract. The authors discuss the potential cellular mechanisms by which alterations of the microbiota could lead to tolerance breakdown, such as modulation of the IgE–basophil axis, alterations in the MyD88 pathway in B lymphocytes and dysregulation in the balance between Th2/Th17 cells.

Advances in sequencing techniques and computation tools have moved forward our understanding of the microbiome's complexities. These advances, discussed by Malla et al., will help catalog the gut microbial community, allowing us to harness its potential use as future diagnostic and therapeutic tools. In summary, the collection of articles in this special issue tackles some of the crucial aspects of gut microbiota in health and disease and highlights an increasing appreciation for the microbiome's contribution to human health.

## Author Contributions

AM and JO-R contributed equally to the organization, writing, and editing of the manuscript. All authors contributed to the article and approved the submitted version.

## Conflict of Interest

AM is one of the inventors of the use of Prevotella histicola for treatment of autoimmune disease, used in this study and the patent is owned by Mayo Clinic Rochester, USA. The technology has been licensed by Mayo Clinic to Evelo Biosciences. AM received royalties from Mayo Clinic (paid by Evelo Biosciences). JO-R serves as a consultant for Symbiotics Biotherapies.
